# News and Miscellany

**Published:** 1902-06

**Authors:** 


### News and Miscellany.

### To the Texas Doctor.

SUCCESS AND GRATITUDE.—We beg to gratefully announce to the medical profession the removal of our offices and laboratories to

105 Chambers St., New York, N. Y., where, with greater space and more extensive facilities, we shall be better enabled to take care of the increasing demand for Ergoapiol (Smith) and Glyco-Heroin (Smith).

---

After having our personal statements as to the unusual efficacy of these preparations in their respective indications verified by hospital and clinical investigations, we sought to interest the physician individually, and the satisfaction of those who have investigated either or both could not be more fully manifested than in the necessity of our new and more commodious quarters.

Indeed none the less is our gratitude to the TEXAS MEDICAL JOURNAL, which has been so frequently mentioned in the communications requesting trial samples, etc., therein showing the large and widespread circulation of so consummate and estimable a journal.

Very sincerely,
MARTIN H. SMITH & CO.

---

The ad. on our front cover is both striking and convincing. It is very striking, because although it may appear in all the other leading medical journals, the *"Red Back"* is the only one that will give it a fitting background, for everything about the Dispensary Tablet package is *red.*

It is convincing because it is true.

Read it again.

---

### The Legal Struggle with Tuberculosis.

"Since the beginning of the last century, no such momentous question in forensic medicine has ever been presented to the general judgment of the Professors of Law and of Medicine, as the one with which now all the civilized nations of the world are confronted, and with which all are now grappling in the question of legislation for its prevention and spread. How far can the ravages of consumption be arrested by legislation? Can any legal enactments or regulations sanctioned by law, arrest its devastating march upon, and its terrible destruction of human life?

"It is the most momentous question of the hour, and last year our best effort was in inaugurating, organizing and rallying all the forces at our command upon this supreme question of medical jurisprudence."—Clark Bell, President Medico-Legal Society, in Inaural Address, January 15, 1902.

Judge Bell writes us May 17 (ult.) regarding the great American Congress of Tuberculosis (held June 3, 4, 5, inst.): "Texas, which has the highest number of members in the Medico-Legal Society (except New York) of any State in the Union, will be away behind, and will not be in the front rank in the Congress, where I wanted to see her. This is due to mistake, I think. I sent to your Governor a list of thirty-odd names of men eligible as delegates from Texas, and asked for their appointment. Texas has only seven delegates named, only three or four of whom can attend. West Virginia sent forty-seven, Missouri twenty-five, new Mexico twenty-three, and most of the other States from seventeen to twenty. Georgia sent twenty from her State Medical Society." Poor Texas, always behind.

